# Poster Session I - A177 PARP1 LACTYLATION IN ESOPHAGEAL SQUAMOUS CELL CARCINOMA LINKS CELL METABOLISM AND GENOME STABILITY

**DOI:** 10.1093/jcag/gwaf042.177

**Published:** 2026-02-13

**Authors:** C Bazin, M Hamilton, V Giroux

**Affiliations:** Cell Biology and Immunology, Universite de Sherbrooke, Sherbrooke, QC, Canada; Cell Biology and Immunology, Universite de Sherbrooke, Sherbrooke, QC, Canada; Cell Biology and Immunology, Universite de Sherbrooke, Sherbrooke, QC, Canada

## Abstract

**Background:**

Esophageal squamous cell carcinoma (ESCC), which accounts for 90% of esophageal cancers worldwide, is one of the deadliest cancers with a 5-year survival rate of only 16% in Canada. Patients with ESCC receive neoadjuvant chemotherapy and/or radiotherapy, which remains ineffective in nearly 50% of patients due to innate or acquired resistance. To explore the mechanisms promoting the emergence of this resistance, proteomic and metabolomic analyses were conducted by our team. These analyses revealed a massive metabolic rewiring after long-term exposure to 5-FU and radiotherapy, notably a significant increase in lactate levels. Lactate has recently been recognized for its role in a new post-translational modification, lactylation, which can alter gene regulation, tumor progression, and macrophage maturation. Interestingly, our mass spectrometry-based lactylome analysis show modulation in the lactylation status of several proteins between ESCC cell lines and an immortalized esophageal epithelial cell line. Interestingly, the protein PARP1 involved in base excision repair (BER), was identified as being lactylated only in ESCC cell lines. While metabolic dysregulation and genomic instability are two hallmarks of cancer, the interconnection between cell metabolism and the response to DNA damage remains largely unknown.

**Aims:**

Thus, it is crucial to understand the functional impact of PARP1 lactylation in ESCC.

**Methods:**

I used co-immunoprecipitations and lactylomes in my study.

**Results:**

By analyzing data available through TCGA, we demonstrated that PARP1 gene expression is significantly increased in esophageal tumors vs normal tissue. By co-immunoprecipitation assays, we confirmed PARP1 lactylation in three ESCC cell lines. We also showed that PARP1 lactylation 1) is maintained upon specific activation of the base excision repair pathway, induced by hydrogen peroxide treatment, and 2) is independent of its PARylation status using PARP and PARG inhibitors. Lactylome analyses highlight several PARP1 lactylation sites including the lysine 105 located in the DNA binding domain suggesting a potential role in DNA repair. To validate lactylation sites and determine the presence of other PTMs (possible competition between them), we will perform IP using PARP1-Trap beads and mass spectrometry analysis. To identify which enzyme is involved in PARP1 lactylation, we will perform siRNA screening of the lactylation “writer” enzymes most frequently referenced in the literature such as CBP, p300, TIP60 and AARS1.

**Conclusions:**

The validation of PARP1 lactylation in ESCC supports the possible link between cell metabolism and the genome stability. Ultimately, this study will provide a better understanding of how metabolic remodeling of cancer cells influences DNA repair, a key factor in tumor progression.

**Funding Agencies:**

CIHR

